# miR-181b as a therapeutic agent for chronic lymphocytic leukemia in the Eμ-TCL1 mouse model

**DOI:** 10.18632/oncotarget.4415

**Published:** 2015-06-10

**Authors:** Antonella Bresin, Elisa Callegari, Lucilla D'Abundo, Caterina Cattani, Cristian Bassi, Barbara Zagatti, M. Grazia Narducci, Elisabetta Caprini, Yuri Pekarsky, Carlo M. Croce, Silvia Sabbioni, Giandomenico Russo, Massimo Negrini

**Affiliations:** ^1^ Università di Ferrara, Dipartimento di Morfologia, Chirurgia e Medicina Sperimentale, Ferrara, Italy; ^2^ Istituto Dermopatico dell'Immacolata, IDI-IRCCS, Laboratorio di Oncologia Molecolare, Rome, Italy; ^3^ Human Cancer Genetics Program and Department of Molecular Virology, Immunology and Medical Genetics, OSU School of Medicine, Ohio State University, Columbus, OH, USA; ^4^ Università di Ferrara, Dipartimento di Scienze della Vita e Biotecnologie, Ferrara, Italy

**Keywords:** chronic lymphocytic leukemia, miR-181b, TCL1, mouse model, gene therapy

## Abstract

The involvement of microRNAs (miRNAs) in chronic lymphocytic leukemia (CLL) pathogenesis suggests the possibility of anti-CLL therapeutic approaches based on miRNAs. Here, we used the Eμ-TCL1 transgenic mouse model, which reproduces leukemia with a similar course and distinct immunophenotype as human B-CLL, to test miR-181b as a therapeutic agent.

*In vitro* enforced expression of miR-181b mimics induced significant apoptotic effects in human B-cell lines (RAJI, EHEB), as well as in mouse Eμ-TCL1 leukemic splenocytes. Molecular analyses revealed that miR-181b not only affected the expression of TCL1, Bcl2 and Mcl1 anti-apoptotic proteins, but also reduced the levels of Akt and phospho-Erk1/2. Notably, a siRNA anti-TCL1 could similarly down-modulate TCL1, but exhibited a reduced or absent activity in other relevant proteins, as well as a reduced effect on cell apoptosis and viability. *In vivo* studies demonstrated the capability of miR-181b to reduce leukemic cell expansion and to increase survival of treated mice.

These data indicate that miR-181b exerts a broad range of actions, affecting proliferative, survival and apoptotic pathways, both in mice and human cells, and can potentially be used to reduce expansion of B-CLL leukemic cells.

## INTRODUCTION

Chronic lymphocytic leukemia (CLL) is a B-cell malignancy with a mature immunophenotype [1, 2]. The disease may present with an indolent asymptomatic course, or, if left untreated, with a rapidly fatal course. Molecular markers have been identified that predict these different clinical courses. Patients with mutated IGHV or few CD38+ or ZAP-70+ B cells generally exhibit the indolent course, whereas patients with CLL cells presenting few or no IGHV gene mutations or numerous CD38+ or ZAP-70+ B cells exhibit an aggressive disease [3-5]. TCL1 - an oncogene whose translocation is the causal factor of T-prolymphocytic leukemia [6] and is overexpressed in many mature T leukemias and B lymphomas of hematological and cutaneous origin [7-10] - is also highly expressed in the aggressive forms of CLL [11].

Multiple pathogenic mechanisms have been suggested as the basis of B-CLL. Chronic antigenic stimulation via the B-cell antigen receptor (BCR) is thought to play an important role. The biologic basis of BCR signaling in CLL has recently been reviewed [12-14]. Sustained engagement of the BCR activates a number of downstream pathways such as NF-kB, AKT and ERK, which contribute to the expansion and survival of malignant cells [1, 15-19]. Other mechanisms important in CLL-cell survival include overexpression of anti-apoptotic proteins such as BCL2, BCL-XL, BAG1, and MCL1 and underexpression of pro-apoptotic proteins such as BAX and BCL-XS; aberration of the ATM/p53 tumor suppressor pathway; deregulation of autocrine cytokines; and interaction with microenvironmental factors such as IL-4, CD40 ligand and accessory cells [20-24].

A better understanding of the molecular pathogenesis of CLL has provided important indications about new therapeutic approaches. A number of clinical studies on novel drugs involved in BCR signaling, such as ibrutinib and idelalisib, inhibitors of the kinases BTK and PI3K, respectively, and ABT-199, a specific BCL2 inhibitor [13, 25-28], have now yielded exciting results in terms of disease response and improved disease-free survival.

The availability of animal models that develop a B-CLL-like disease may help the testing of additional unexplored therapeutic approaches. A recent survey of mouse models indicated that the Eμ-TCL1 transgenic and 13q14 deletion models develop leukemias that closely resemble human CLL [29]. Specifically, the transgenic mouse Eμ-TCL1 develops a leukemia characterized by clonal expansion of cells with a B220+/CD5+ immunophenotype, non-mutated IGHV, increased proliferation and enhanced Akt phosphorylation, which may well represent an aggressive form of CLL [30-33]. In the present work, we used the Eμ-TCL1FL transgenic mouse model [31] to determine whether microRNAs (miRNAs) could represent novel therapeutic tools for the treatment of B-CLL.

miRNAs are a class of highly conserved small noncoding RNAs that regulate post-transcriptional gene expression [34]. A single miRNA may control the expression of hundreds mRNA targets. Just as miRNAs may control a variety of crucial functions related to normal cell growth, development and differentiation, so their dysregulation is associated with pathological conditions [35]. The first evidence of miRNA involvement in human cancer came from a study on CLL [36]. Subsequent studies unraveled miRNA signatures that could be associated with prognosis and progression in B-CLL or with cytogenetic subgroups [37-40], thus denoting an important role of miRNAs in CLL pathogenesis. Some of these miRNAs could target or be regulated by tumor suppressors or oncogenes (reviewed by [41, 42]). For example, miR-15 and miR-16, which are deleted or down-regulated in the majority of B-CLL patients, can modulate genes involved in proliferation and survival pathways. Among these, Bcl2 was demonstrated to be a direct target [43, 44]. miR-34a is activated by p53 and miR-155 is induced by STAT3 [45]. miR-181 and miR-29 could down-regulate TCL1, Mcl1, Bcl2 and Bcl2L11 [46-48].

These studies suggest that miRNAs have a potential use in targeting survival pathways to elicit a therapeutic effect against CLL cells [49-51]. Here, we explored how the enforced expression of miR-181b, which is down-regulated in human CLL [39, 40] and has been associated with disease progression [38, 52], could affect the viability of leukemic cells developing in the Eμ-TCL1 model.

## RESULTS

### miR-181b down-regulates TCL1 and reduces viability in human and mouse malignant B cells

It was previously demonstrated that miR-29b and miR-181b could down-modulate TCL1 protein expression in HEK293 cells [46], but the same results have not been yet reported in B cells. This represents a potentially relevant finding since these miRNAs were shown to be underexpressed in CLLs, in particular in the aggressive forms where TCL1 is highly expressed [11]. Unfortunately, because no CLL cell line is available, we had to investigate B cells, such as RAJI and 697, which have high TCL1 expression [6, 53] but originate from B-cell malignancies other than CLL.

Following transfection of miRNA mimics of the miR-181 and miR-29 families, we assessed the TCL1 protein level. A scrambled RNA oligo was used as a negative control to normalize data. In general, all miRNAs could down-modulate TCL1 protein, albeit with differences between the two cell lines (Supplementary Figure S1A-B). After averaging all data, miR-181b emerged as being the most consistent in reducing TCL1 protein levels (*P* < 0.0005) (Supplementary Figure S1C). Specificity of miR-181b activity was further confirmed by anti-miR-181b, which induced an increment in the TCL1 protein level (Supplementary Figure S2).

**Figure 1:**
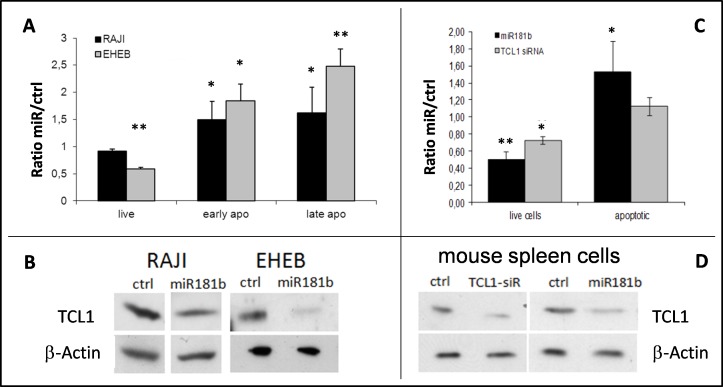
Viability and apoptotic effects following mir-181b enforced expression in human RAJI and EHEB cells and in mouse TCL1-tg leukemic splenocytes **A.** RAJI (black bars) and EHEB (gray bars) cells were transfected with miR-181b. Viability and apoptosis were assessed by Annexin V and PI staining analyzed by flow cytometry (see representative examples in Supplementary Figure 3). The reported values represent the miRNAs versus negative control (ctrl) cell count ratio from 3 independent experiments. **B.** Western blot analyses showing the TCL1 down-regulation following miR-181b transfection (in RAJI cells, the shown lanes were from the same blot, but not originally next to each other). **C.** Apoptosis and viability of mice tumor splenocytes were assessed through flow cytometry analysis. Mean values of six independent experiments were expressed as the ratio of miR-181b (black) versus control transfected cells and compared with those obtained by using anti-TCL1 siRNA (gray) (*P < 0.05 **P < 0.01). **D.** TCL1 Western blot analysis of splenocytes after 72 h of transfection with anti-TCL1 siRNA or miR-181b or control (ctrl). (*P < 0.05 **P < 0.01).

Having identified miR-181b as the most consistent regulator of TCL1 expression among those tested, we assessed its effects on cell viability. Following transfection of miRNA mimics, we measured apoptosis and cell viability by fluorescence-activated cell sorting (FACS) analysis (Figure 1A-1B and Supplementary Figure S3). In RAJI cells, miR-181b induced a 1.5- and 1.6-fold increase in early and late apoptosis, respectively. Moreover, in EHEB cells, an Epstein-Barr virus-immortalized cell line established from a CLL patient [54], miR-181b induced a pronounced reduction of TCL1 protein (> 80%) accompanied by a significant increase in apoptosis (2.5- and 1.8-fold increase in early and late apoptosis, respectively) and a reduction in the proportion of live cells.

We next analyzed the expression of miR-181b and TCL1 protein levels in cells isolated from the spleen of individual 12- to 16-months old TCL1-tg mice with overt leukemia (Supplementary Figure S4A-B). Similarly to CLL patients [46], an inverse correlation between miR-181b expression and TCL1 protein levels was observed in TCL1-tg leukemic splenocytes (Supplementary Figure S4C), suggesting the existence of miR-181b regulation of TCL1 protein in these cells as well. We confirmed this hypothesis by showing the ability of miR-181b to down-regulate TCL1 protein similarly to anti-TCL1 siRNA in TCL1-tg leukemic splenocytes (Figure 1D). Notably, however, miR-181b reduced cell viability and increased apoptosis to a much higher extent than did anti-TCL1 siRNA (Figure 1C). miR-181b reduced the viability of mouse malignant cells to 50% of that of controls (*P* < 0.01) and resulted in a 1.5-fold increase in apoptosis (*P* < 0.05). This finding suggested that the biological effects of miR-181b were mediated by mechanisms other than, or in addition to, TCL1 down-regulation.

### miR-181b modulates several pathways involved in CLL

**Figure 2:**
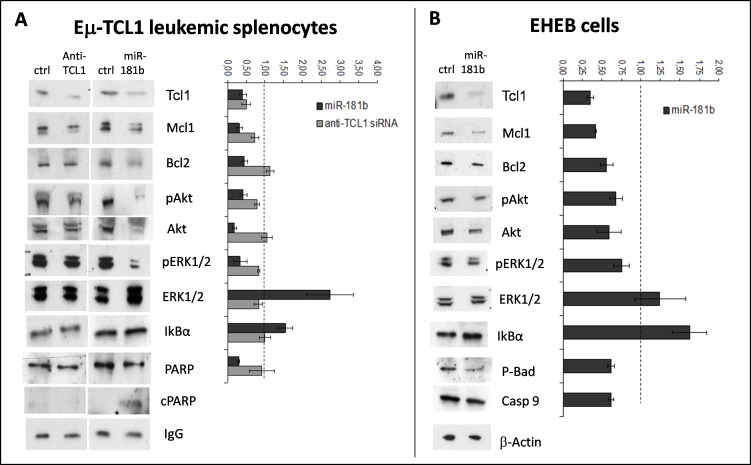
miR-181b modulates key factors involved in CLL **A.** Immunoblotting analysis of Mcl1, Bcl2, phospho-Akt (p-Akt), Akt, phospho-ERK1/2 (p-ERK1/2), ERK1/2, IkBα, and PARP (cleaved form, cPARP) in leukemic splenocytes after transfection with miR-181b or anti-TCL1 siRNA or negative control (ctrl). IgG served as the loading control. The densitometric ratio between miR181b (black bars) or anti-TCL1 siRNA (gray bars) and the respective control (dashed line) is shown at right. **B.** The same immunoblotting analyses were performed in EHEB cells, except that caspase-9 and phospho-BAD (p-BAD) were analyzed instead of PARP. The densitometric ratio miR-181b/ctrl is shown at right. All experiments were repeated three times and produced similar results.

To investigate the molecular basis of the difference between miR-181b and anti-TCL1 siRNA on viability and apoptosis, we analyzed the effects of these small RNAs on key proteins involved in CLL. We quantified protein levels by Western blotting in mouse leukemic splenocytes transfected with miR-181b or anti-TCL1 siRNA (Figure 2A). Experiments were performed in triplicate to confirm reproducibility of data.

As shown earlier, miR-181b could efficiently down-regulate TCL1 protein similarly to anti-TCL1 siRNA. Conversely, Mcl-1 and Bcl2, two anti-apoptotic factors, were both down-modulated by miR-181b (about 70% and 50%, respectively), whereas anti-TCL1 siRNA induced only a slight reduction in MCL1 (about 20%) and had no effect on BCL2. The activation of apoptosis was confirmed by analysis of Poly (ADP-ribose) polymerase (PARP): a 70% reduction of the intact form and the appearance of the 85-kD fragment of cleaved PARP were seen only in the miR-181b transfected cells.

Akt and MAPK pathways were also analyzed after miR-181b or anti-TCL1 siRNA transfection. miR-181b induced a 60-70% reduction in Akt and phospho-Akt levels; conversely, anti-TCL1-siRNA did not affect Akt levels and we detected only a slight p-Akt reduction, which was likely due to the down-regulation of TCL1, a well-known activator of Akt [55]. We also found a marked reduction of phospho-ERK (65%), despite there being an increase in ERK protein in miR-181b transfected cells. Conversely, no significant effects were induced by anti-TCL1 siRNA. Thus, compared with anti-TCL1-siRNA, miR-181b has a wider capacity to regulate proteins implicated in cell survival, which could explain the major effects of the miRNA on cell apoptosis and viability in TCL1-tg mouse leukemic cells.

We studied the same pathways in human EHEB cells (Figure 2B). In essence, we found the same pattern of modifications. In these cells, in order to monitor apoptosis at the molecular level, we examined the Bcl-2-associated death promoter (BAD) and caspase-9 instead of PARP [56-59]: the reduced level of p-BAD, a target of p-Akt, and the increase in caspase-9 cleavage, indicated the induction of molecular mechanisms responsible for the apoptosis shown in Figure 1A.

Thus, *in vitro* results in both mouse and human cells showed that enforced miR-181b expression exerts a broad range of actions, affecting proliferative, survival and apoptotic factors, and indicated the appropriateness of the TCL1-tg mouse model for testing miR-181b as a therapeutic agent against mouse leukemic cells *in vivo*.

### miR-181b slows leukemia in the CLL mouse model

With a 100% penetrance at 12 months of age, the TCL1-tg mouse transgenic model is highly predisposed to the development of a leukemia characterized by cell immunophenotype and behaviors that are similar to human CLL [30, 31]. However, because of the intrinsic heterogeneity of leukemic lines derived from different mice, we used a syngeneic transplantation approach [60] to perform *in vivo* experiments in more uniform experimental conditions.

**Figure 3:**
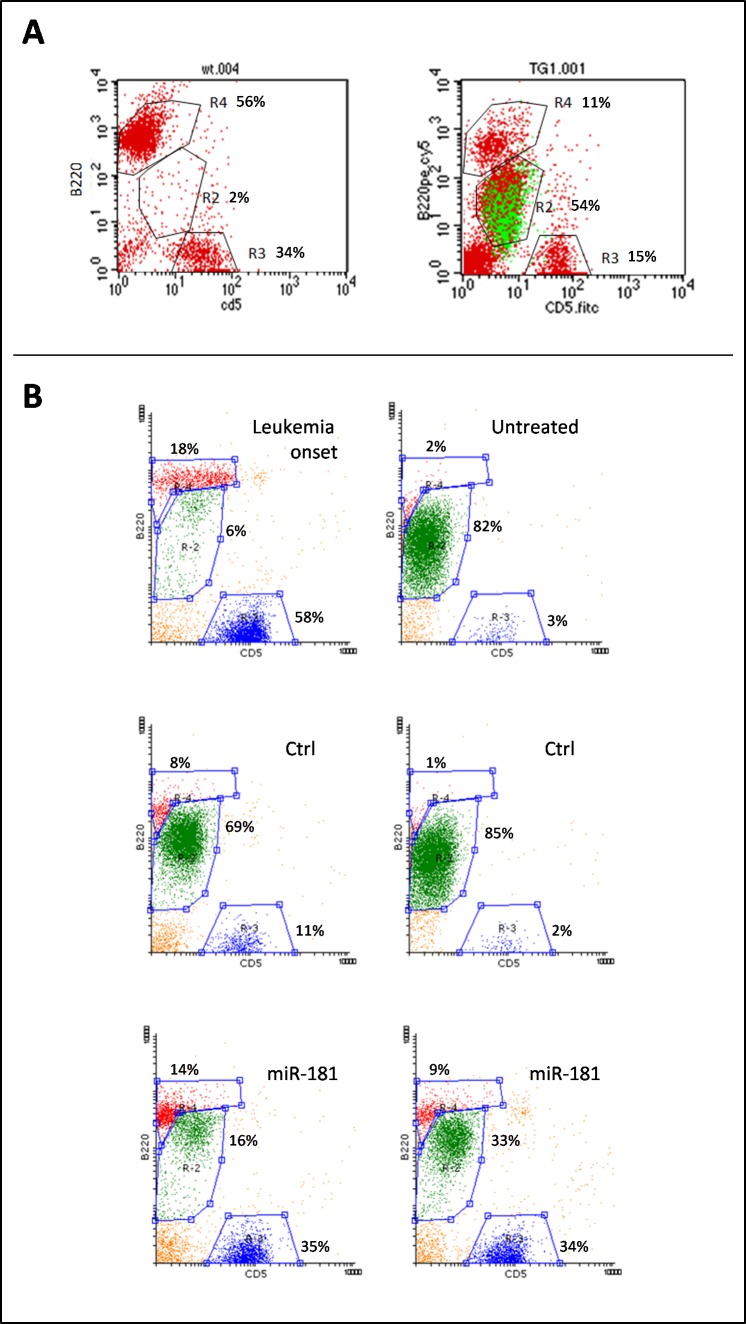
Leukemic expansion (LE) monitored by B220+/CD5dim immunostaining Peripheral blood cells were characterized for the expression of B220 and CD5 surface markers. **A.** The expansion of B220+/CD5dim leukemic cells can be seen in the R2 gate area, abundantly in the TCL1-tg mouse and virtually absent in the wt mouse; moreover, intracellular staining for human Tcl1 protein revealed identity between the Tcl1+ cells (green) and the B220+/CD5dim subpopulation of gate R2, indicating this to be the leukemic clone. Few cells that appeared as Tcl1- in this pool might be the result of permeability failure. **B.** Representative plots of pre-treatment leukemia onset and post-treatment untreated mice, negative control (ctrl) or miR-181b-treated mice. LE was calculated as the number of leukemic cells (gate R2, green dots) relative to total lymphocytes: R2 + R3 (normal T cells, blue dots) + R4 (normal B cells, red dots). The images show clear differences between miR-181b-treated mice and controls.

Syngeneic transplants were performed in 6- to 8-week-old FVB wild-type (wt) mice by intraperitoneal (i.p.) injection of 5×10^5^ splenocytes, collected from two different diseased TCL1-tg donors (specifically, the F3 and F15 shown in Supplementary Figure S4), when they were both 14 months old and spleen leukemic cells were more than 80% of total white cells. Leukemia expansion (LE), measured as the fraction of malignant cells to total lymphocytes, was monitored in transplanted mice every 2 weeks by peripheral blood mononuclear cell (PBMC) immunophenotyping, through flow cytometry analysis [30, 31]. Lymphocytes were sorted on the basis of physical parameters and subpopulations were identified as follows: leukemic B cells were B220+/CD5dim (R2 region), normal B lymphocytes were B220+/CD5- (R4 region) and T lymphocytes were B220-/CD5+ (R3 region) (Figure 3). B220+/CD5dim cells were confirmed to originate from transplanted transgenic leukemic cells by staining for human TCL1 protein (Figure 3A). Despite the relatively low expression of CD5, leukemic cells were discernible from normal B cells without difficulty because of weaker B220 staining.

Mice were enrolled for treatment when LE values ranged from 0.05 to 0.2 (i.e. leukemic cells representing 5-20% of peripheral blood lymphocytes), which were reached at 8 weeks after transplantation. At this stage, mice were randomized into three groups (average LE in each group = 0.1): 9 mice were treated with miR-181b, 9 mice were treated with scrambled negative control and 15 mice were left untreated. In accordance with preliminary analyses (Supplementary Figure S5), mice were treated with 80 μg of miR-181b twice a week for three consecutive weeks. We used the cationic polymer polyethylenimine (PEI) as vehicle for mimic systemic administration. This vehicle was shown to improve siRNA delivery to several organs [61] and we also confirmed a good efficiency of delivery to the spleen and uptake from splenic cells, compared to mimic alone (Supplementary Figure S5). A clear reduction of the leukemic cell fraction was detectable in the samples derived from mice treated with miR-181b mimics (representative plots are shown in Figure 3B). A reduction of normal B population is notable in peripheral blood in the presence of high LE, which was due to the replacement of normal with malignant cells [30], rather than to detection failure or side-effects related to miR-181b treatment (Supplementary Figure S6).

**Figure 4:**
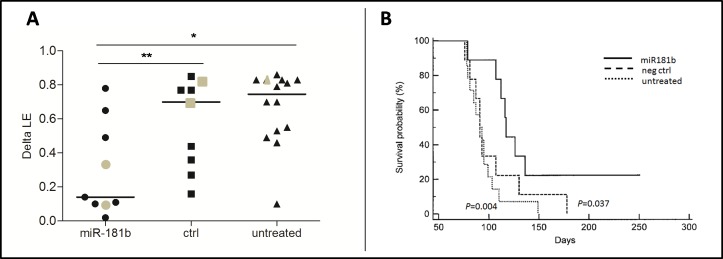
Administration of miR-181b to TCL1-tg mice induces delay in leukemic expansion (LE) and increases survival **A.** Differences between LE measured three days before the beginning of treatment and three days after the end of treatment (delta LE) for miR-181b (*n* = 9), negative control (ctrl)-treated (*n* = 9) and untreated (*n* = 15) mice are reported in the scatter plot. The disparity in delta LE values between miR-181b-treated mice and negative control-treated or untreated animals is statistically significant (*P < 0.01; **P < 0.05). Grey dots identify the animals shown in Figure 3B. **B.** Kaplan-Meier survival curves for the three groups, miR-181b-treated (*solid line*), negative control (neg ctrl)-treated (*dashed line*) or untreated mice (*dotted line*) were also assessed. The miR-181b group shows significantly better survival (median survival 117 days) than the negative control group (median survival 91 days; *P* = 0.037) and the untreated group (median survival 92 days; *P* = 0.004). No significant differences between the negative control and untreated groups were observed (*P* = 0.49).

For each mouse, we assessed the difference between LE measured before and after treatment (delta LE). As illustrated in Figure 4A, the median delta LE was 0.14 after miR-181b treatment, whereas it was 0.70 in the control group and 0.75 in the untreated mice. The differences in delta LE values between miR-181b-treated and untreated mice or between miR-181b-treated and negative control-treated mice were both statistically significant (*P* = 0.005 and *P* = 0.04, respectively), whereas the differences between negative control-treated and untreated mice were not significant (*P* = 0.30). Notably, response to miR-181b treatment was comparable in leukemias derived from the two donors (F3 and F15), despite the different Tcl1 and miR-181b endogenous levels (Supplementary Figure S4A-B).

Finally, we performed a survival analysis of treated and untreated mice. In accordance with institutional animal care guidelines, a humane end point was defined at LE = 0.85 and the animals were euthanized when they reached this clinical stage. Survival data were collected and reported in a Kaplan-Meier function (Figure 4B). Differences between curves were analyzed by using the log-rank (Mantel-Cox) test. Survival in the miR-181b treated group was significantly increased for both untreated (*P* = 0.004) and negative control-treated (*P* = 0.037) groups. No significant divergence was found between negative control-treated and untreated mice (*P* = 0.49). These data indicate that miR-181b is efficient in reducing leukemic cell expansion, resulting in prolonged survival.

## DISCUSSION

In this study, we took advantage of the TCL1-tg mouse model characterized by the development of a leukemia whose features are similar to those of the aggressive form of human CLL [29, 31] to test the therapeutic efficacy of miR-181b.

This miRNA was chosen because it is underexpressed in aggressive CLLs and TCL1 was shown to be one of its targets [46]. Our results showed that miR-181b could down-regulate TCL1 in malignant B-cell lines with high expression of endogenous TCL1. From our analyses, miR-181b appeared to be the strongest TCL1 inhibitor among members of the miR-181 or miR-29 families. However, the simple down-regulation of TCL1, achieved through the use of anti-TCL1 siRNA, did not achieve the level of apoptosis induced by miR-181b in EHEB cells and mouse leukemic cells derived from the TCL1-tg mouse, indicating the existence of relevant targets other than TCL1 that are important in mediating biological effects. Indeed, miR-181b shares the down-regulation of TCL1 with anti-TCL1 siRNA, but the effects on MCL1, BCL2, Akt and p-Erk were mainly or only induced by miR-181b.

In addition to *in vitro* experiments, we performed *in vivo* studies by using the TCL1-tg mouse model. miRNA delivery in *in vivo* experiments represents a crucial hurdle because of the degradation of miRNA by nucleases and its low cellular uptake. In this study, we used a simple procedure for effective delivery of miRNAs to the spleen. This was an essential requirement, as TCL1-induced leukemia is characterized by splenomegaly, suggesting that the spleen harbors proliferating centers from which circulating leukemic cells originate. We used the cationic polymer PEI, a vehicle currently being used in phase I and II clinical trials [62]. The use of PEI was necessary to significantly increase the efficiency of the delivery of miRNA mimics to the spleen, which was otherwise negligible in its absence [61].

The results of *in vivo* experiments did not attain leukemia regression, but significantly slowed the disease, thus demonstrating an *in vivo* anti-leukemic activity of miR-181b that was clearly detectable after 3 weeks of treatment. Treated mice showed reduced leukemia growth and prolonged survival compared with control groups, producing a proof of principle that miR-181b has a measurable therapeutic activity against CLL cells. These findings, in line with other studies in which synergistic activity of miR-181b with fludarabine was observed in human primary CLL cells [63], add novel additional evidence for a potential role of miR-181b as a therapeutic agent in CLL.

At least two explanations can be suggested for the lack of leukemia regression induced by miR-181b as a single agent: (i) The miRNA could not induce death of leukemic cells because it was incompletely effective; and (ii) the approach was incomplete and a number of leukemic cells escaped delivery.

In the first case, after miR-181b restoration, molecular analyses revealed a broad pattern of inhibition on important pathways involved in CLL. We found, in agreement with previous reports, a decrease in TCL1, Mcl1 and Bcl2 protein levels [48, 52, 63, 64], both in malignant cells isolated from the spleen of TCL1-tg mice and in the human CLL cell line EHEB. In addition, because of the known role of Tcl1 in Akt activation [55], miR-181b and anti-TCL1 siRNA were both likely responsible for the down-regulation of p-Akt, which was in turn responsible for the reduction of p-Bad. The down-regulation of non-phosphorylated Akt protein was induced by miR-181b, but not anti-TCL1 siRNA, suggesting that either a direct or an indirect targeting by miR-181b had occurred. Moreover, miR-181b was able to modulate the MAPK pathway through a marked down-modulation of phosphorylated active ERK1/2, a key factor in promoting proliferation signals through the MAPK pathway, which contributes to leukemia and is involved in drug resistance [65-67]. ERK1/2 was not directly targeted by miR-181b, as shown by the unaffected level of non-phosphorylated ERK, but the cross-talk between Ras/MEK/ERK and PI3K/AKT pathways, reported in many tumors [68-70], suggested that the down-modulation of Akt could also influence Erk phosphorylation. Overall, the pathways affected by miR-181b are highly relevant for CLL pathogenesis, and the simultaneous inhibition of Akt and MAPK pathways, together with the repression of anti-apoptotic proteins such as TCL1, Bcl2 and Mcl1, appears to represent a wide and highly effective panel of targets for achieving a strong therapeutic effect, as shown by the fact that several of these pathways are targeted by some of the newly available drugs against CLL [71].

In the second case, improved efficiency and specificity in the delivery approach might enhance miR-181b anti-leukemic activity. Approaches that could improve these parameters already exist and need to be experimentally assayed. Here, we found that PEI significantly improves the delivery of miR-181b to the spleen in comparison with mimic alone, but margins of progress may still exist. The use of liposomes or nanoparticles conjugated with specific antibodies may increase the specificity for better targeting of CLL cells and represent feasible approaches [72-74].

The use of miR-181b against leukemia that develops in the Eμ-TCL1 mouse model may present a potential drawback, as it may be argued that the anti-leukemic effect of miR-181b is effective only against the leukemic cells of this model because they are driven by TCL1, which is a miR-181b target. However, some counterarguments can be suggested. First, we performed experiments by using anti-TCL1 siRNA, which, theoretically, should have produced the same biological results, if the observed effects were all mediated through TCL1 down-regulation. This was not the case: miR-181b induced stronger apoptosis than did anti-TCL1 siRNA. As mentioned earlier, multiple survival pathways were affected by miR-181b, but not by anti-TCL1 siRNA, which may explain the different actions on apoptosis and viability. In addition, miR-181b was similarly effective in mice transplanted with leukemic cells with either high or low Tcl1 levels. Finally, miR-181b has a pathogenic role in CLL: it is down-regulated in human CLL [39, 40] and its down-regulation has been associated with disease progression [38, 52]. Hence, these arguments make it conceivable that the therapeutic effects observed following the restoration of miR-181b represent a general mechanism that is not limited to the leukemic cells of the Eμ-TCL1 model.

In summary, although the first evidence of involvement of a miRNA in human malignancies was reported in CLL about 13 years ago, this is the first *in vivo* preclinical study that provides evidence of a miRNA with potential therapeutic activity against CLL cells. A number of additional miRNAs are now known to be dysregulated in CLL, suggesting that several could and should be tested for their therapeutic activities. Additionally, the report of the synergistic activity of miR-181b with fludarabine [63] suggests an important field of studies for investigating the ability of miRNAs to improve the efficacy of traditional therapeutic approaches, with minimal side effects on normal B and T cells.

## MATERIALS AND METHODS

### Animal model and *in vivo* experiments

The Eμ-TCL1FL transgenic mouse (TCL1-tg) was produced and characterized at Ohio State University as previously described [31]. Syngeneic FVB wt mice were obtained from Charles River Laboratories. Mice had *ad libitum* access to water and a pellet diet. The animal room was maintained at 23°C on a 12-h light/12-h dark cycle. Syngeneic transplants were performed in 6- to 8-week-old FVB wt mice by i.p. injection of 5×10^5^ tumor splenocytes, collected from diseased TCL1-tg donors. LE was monitored every 2 weeks by PBMC immunophenotyping, through flow cytometry analysis. Mice were enrolled for treatment when the disease reached between 0.05 and 0.2 LE values. Prior to administration, miR-181b or negative control mimics (Axolabs, Germany) were assembled in a complex with *in vivo* jetPEI (Polyplus-Transfection SA, France), using a nitrogen-to-phosphate ratio of 8, as recommended by the manufacturer. The i.p. injections were administered twice a week for 3 weeks, using 80 μg of miR-181b each. At the end of the treatments, LE was measured and the delta value was calculated for each mouse as the difference between LE before and after treatment. All manipulations with the mice were conducted according to directive 2010/63/EU of the European Parliament and of the Council and approved by the local ethical committee for animal experimentation. The end of the experiments was reached when mice were euthanized just before the appearance of clinical signs, such as weight loss; interference with locomotion, grooming and vital physiological functioning; apathy; or death.

### Cell cultures and transfections

The human TCL1-expressing B-cell lines 697, RAJI and EHEB were maintained in RPMI 1640 medium, supplemented with 10% fetal bovine serum. Mouse splenocytes were freshly isolated from diseased mice, using the following procedure: spleens were explanted from euthanized mice and placed in cold phosphate-buffered saline (PBS) solution. Cell suspension was obtained by squeezing spleen between two slides and washing with PBS. Erythrocytes were degraded by using ammonium chloride (0.8%) with EDTA (0.1 mM) (Sigma). White cells were washed, passed through a 70-μm filter and counted. Depending on the experimental procedure, the B-cell pool was further purified by using B220-conjugated magnetic beads (Invitrogen) following the manufacturer's instructions. Leukemic splenocytes were cultured in 48 multi-well plates, using RPMI 1640 medium supplemented with 10% fetal bovine serum, 10 mM HEPES buffer and 1 mM Na pyruvate. Transient transfections were performed with 100 nM pre-miR (Ambion) or single-stranded mimic (IDT) or 20 nM anti-TCL1 siRNA (Dharmacon), or scrambled negative controls, diluted in Opti-MEM serum-free medium (Gibco) and complexed with INTERFERin transfection reagent (Polyplus), following the manufacturer's instructions. Cells were harvested 72 h after transfection and were either lysed in RIPA buffer for Western blot analysis or stained for FACS analysis.

### Western blot analyses

Equal amounts of protein were electrophoresed in SDS-polyacrylamide gels and blotted onto nitrocellulose filters (Bio-Rad). Membranes were blotted overnight at 4°C with primary antibodies. The employed antibodies were the following: anti-human TCL1 (27D6) from Areta International; Akt (cat. 9272), phospho-Akt (cat. 4058), ERK (cat. 9102), phospho-ERK (cat. 9101), Bcl-2 (cat. 3498), Mcl-1 (cat. 5453), and phospho-Bad (cat. 9295) from Cell Signaling Technology; PARP (cat. NB100-111) from Novus Biologicals; caspase-9 (cat. RB1205P1) by Neo Markers; and IkBα (cat. sc-371), β-actin (cat. sc-130656), anti-mouse IgG-HRP (cat. sc-2005) and anti-rabbit IgG-HRP (cat. sc-2030) from Santa Cruz Biotechnology. For immunodetection, Pierce ECL plus or Super Signal West Femto chemiluminescent substrates (Thermo Scientific) and GE Healthcare films were used. Protein bands were quantified by scanner densitometry (GS-710, Bio-Rad) and analyzed with Quantity One software (Bio-Rad). TCL1 OD values were first normalized on β-actin expression and then on control (ctrl) TCL1/β-actin ratio.

### Flow cytometry

Flow cytometry was performed on FACSCalibur (BD Bioscience), using CellQuestPro software (BD Bioscience). For apoptosis analysis, cell staining was performed as follows: 72 h after transfection, cells were incubated for 15 min with FITC-anti-mouse CD5 antibody (BD Bioscience), washed and suspended in 100 μL of PBS solution containing 1.25 μL of APC-conjugated Annexin V (Ax) (eBioscience) and incubated for 15 min in the dark; 500 μL of PBS and 2 μg/mL of propidium iodide (PI) (Sigma) were added and cells were immediately analyzed. CD5dim cells were identified as the leukemic population and gated in an Ax/PI plot. Early apoptotic cells were identified by Ax+/PI- staining, late apoptotic by Ax+/PI+ staining and live cells by Ax-/PI- staining. Immunophenotyping of PBMCs was performed as follows: blood samples were collected from the retro-orbital plexus of anesthetized mice and placed in a tube containing 0.5 M EDTA as an anticoagulant. Erythrocytes were broken by treatment with ammonium chloride (0.8%) and EDTA (0.1 mM) (Sigma). PBMCs were incubated with FITC-conjugated anti-mouse CD5 and PeCY5-conjugated anti-mouse B220 surface markers (BD Bioscience) for 15 min at room temperature, washed and analyzed. Lymphocytes were recognized by physical parameters and gated on a B220/CD5 plot. Leukemic cells were identified as B220+/CD5dim cells (R2 region), normal B lymphocytes as B220+/CD5- cells (R4 region) and T lymphocytes as B220-/CD5+ cells (R3 region). LE was evaluated as the ratio of leukemic cells relative to total lymphocytes LE = R2/(R2+R3+R4). For TCL1 staining, after incubation with anti-mouse B220 and CD5, PBMCs were stained with anti-human TCL1 for 1 h by using APC-conjugated antibody (eBioscience) diluted in a solution containing 0.1% saponin, and then washed and analyzed. Transgenic TCL1+ cells were gated and visualized on a B220/CD5 plot (green population), along with total lymphocytes (red).

### RNA extraction and real-time PCR

Total RNA was extracted from B220+ cells of wt or TCL1-tg spleens, using TRIzol (Invitrogen), according to the manufacturer's instructions. miR-181b retro transcription and amplification was obtained through the TaqMan MicroRNA Assay, TaqMan MicroRNA Reverse Transcription kit and TaqMan Universal MasterMix (Life Technologies), following the manufacturer's recommendations. qPCR was performed by using the ABI PRISM 7000 Sequence Detection System (Life Technologies) and fold-change values were calculated by using the ΔΔCt method.

### Statistical analysis

All data reported as histograms are expressed as mean ± SD; the *P*-values, calculated by t-test, were considered to be statistically significant when less than 0.05. A nonparametric model was used for correlation analysis (Spearman correlation coefficient) for LE comparison among mice (Mann-Whitney test) and for Kaplan-Meier survival analysis (Mantel-Cox test). For all statistical procedures, Graphpad Prism 6.0 software products were used for data analysis.

## SUPPLEMENTARY FIGURES


