# Supplementary material for: miR-181b as a therapeutic agent for chronic lymphocytic leukemia in the Eμ-TCL1 mouse model
Source: Oncotarget. 2015 Jun 10;6(23):19807–18. doi: 10.18632/oncotarget.4415 (PMC4637322; doi:10.18632/oncotarget.4415)
Supplement: Supplementary file 1 [file oncotarget-06-19807-s001.pdf]

miR-181b as a therapeutic agent for chronic lymphocytic leukemia in the Eμ-TCL1 mouse model

Supplementary Material

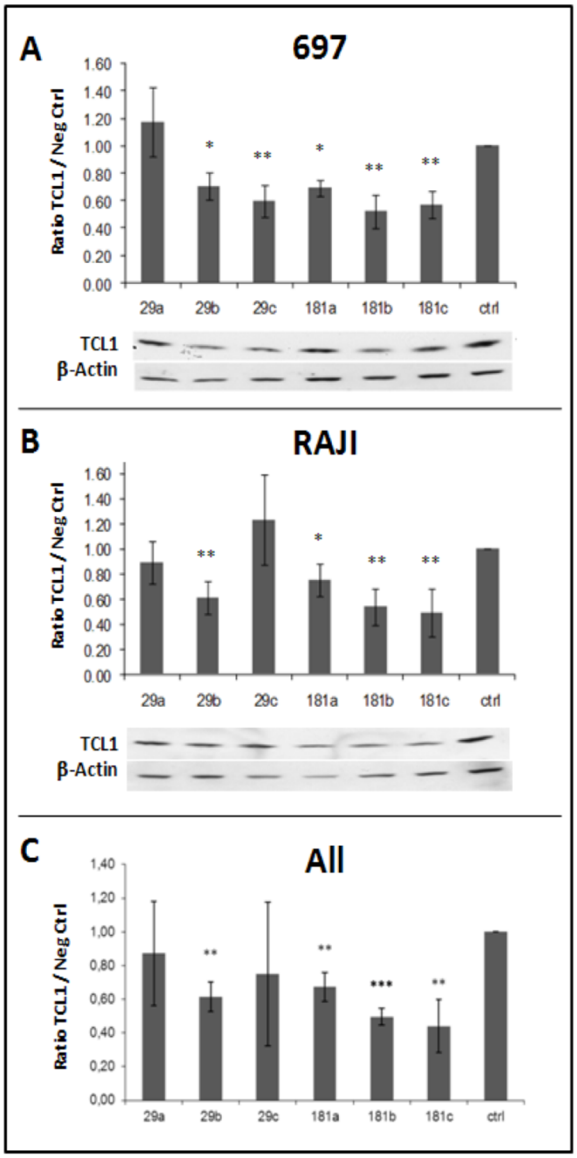

Figure S1. Analysis of TCL1 modulation by members of the miR-29 or miR-181 families in human B-cell lines. TCL1 and β-actin expression were evaluated by Western blot analyses. The graphs represent the average ratios between TCL1 and β-actin OD values from three independent experiments. For each experiment, all ratios were further normalized on ctrl TCL1 / β-actin OD ratios. Transfection experiments were performed in the human B-cell lines 697 (**A**) or RAJI (**B**). Representative experiments are shown. Panel **C** represents the summary of results from all six experiments. P-values were calculated by t-test. **P*<0.05; ***P*<0.005; ****P*<0.0005.

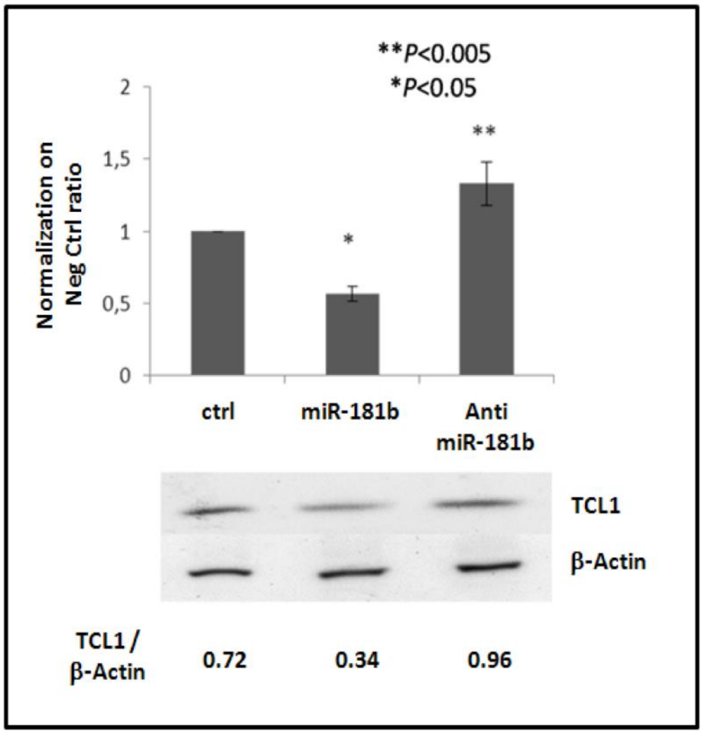

Figure S2. **TCL1 protein levels are modulated by miR-181b mimics and anti-miR-181b oligos in human RAJI B-cells.** miR-181b mimics induce the down-modulation of TCL1, whereas anti-miR-181b oligonucleotides promote its up-regulation. Data under the image is the ratio between TCL1 densitometric OD and β-actin OD. The graph bars represent data normalized on TCL1 / β-actin ratio in control (ctrl) samples from three independent experiments.

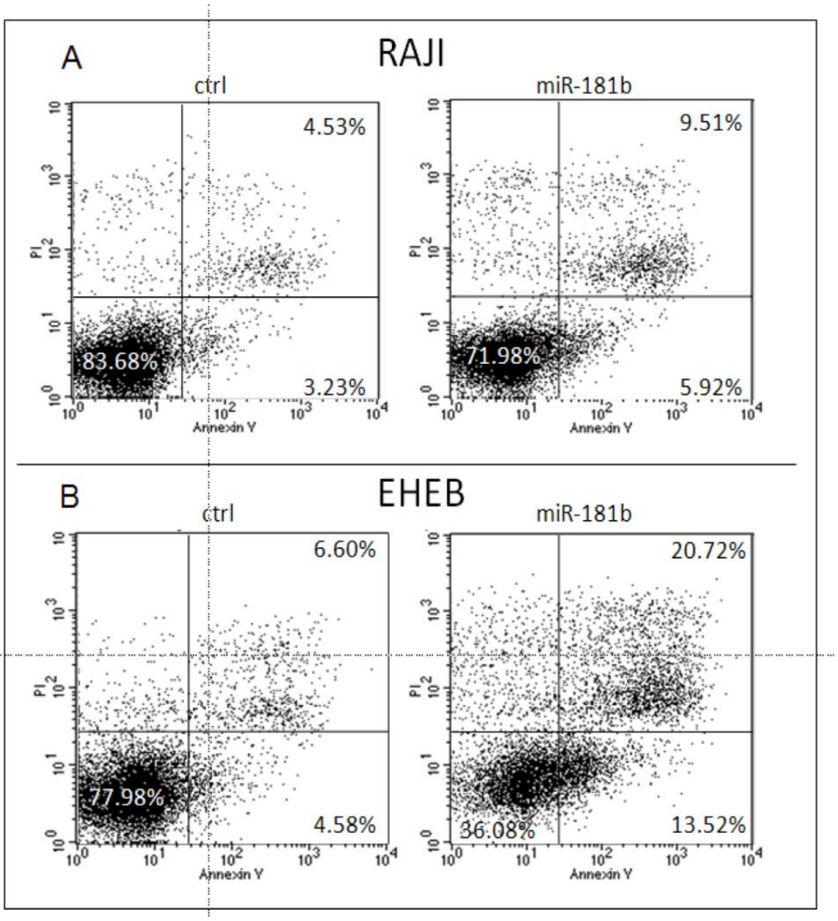

Figure S3. miR-181b induces apoptosis in human B-cell lines. Representative plots are shown for human B-cell lines treated with miR-181b or ctrl for 72hrs and analyzed for apoptosis induction by flow cytometry and AnnexinV/PI staining. Cells undergoing apoptosis are visible in the right lower window (early apoptosis, Annexin V + / PI -) and in the right upper window (late apoptosis, Annexin V + / PI +). An increase in apoptotic cells are readily detectable following miR-181b transfection compared to ctrl in both RAJI (**A**) and EHEB cells (**B**).

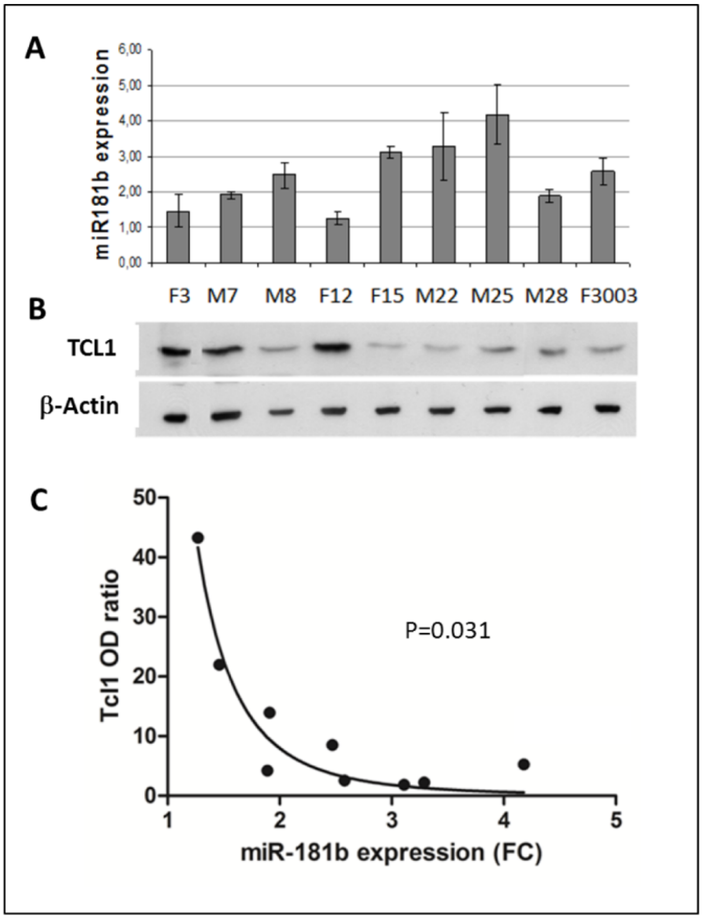

Figure S4. Correlation between endogenous miR-181b and TCL1 levels in leukemic cells from various transgenic mice. (**A**) miR-181b levels in B cells isolated from spleen of leukemic TCL1-tg mice between 12 and 16 months, were obtained by quantitative PCR. Fold-change values (FC) for miR-181b expression were calculated with respect to the mean value of wt. (**B**) Tcl1 protein levels in the same leukemic mice were assessed by Western blot analysis, normalized for β-actin and reported as the OD ratio relative to the less abundant sample. (**C**) For each leukemic mouse, the miR-181b FC value and the Tcl1 level were graphed. The Spearman correlation coefficient revealed an inverse correlation between the two variables (r=-0.7333; *P*=0.031).

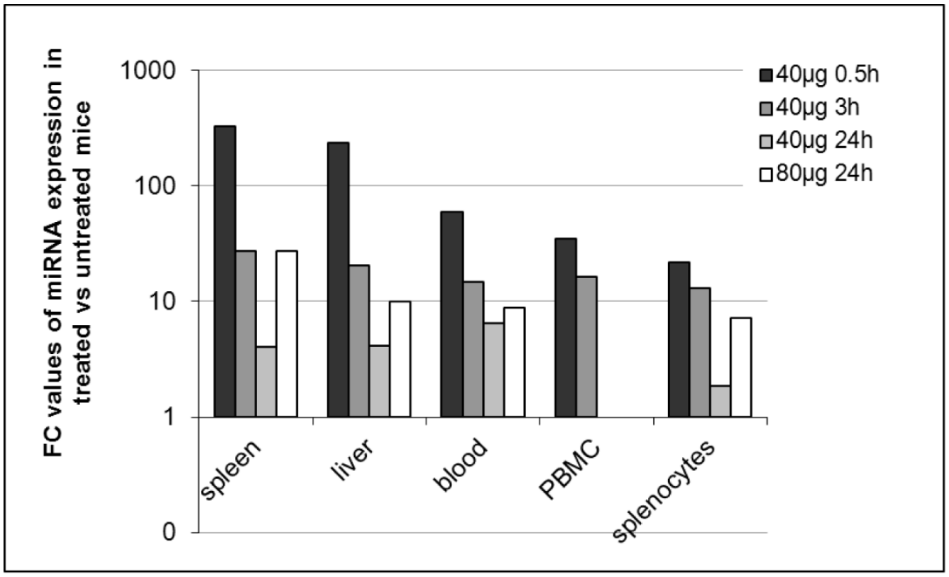

Figure S5. miRNA injection produces an increased level in organs/tissues of interest. Wt mice were injected i.p. with 40 µg or 80 µg of miR-181b mimics molecules complexed with jetPEI. Organs were explanted at the indicated time points and miRNA levels measured by qPCR. Increased levels of miR-181b in comparison to untreated mice are reported as fold-change (FC) values in a logarithmic scale. The increase of miR-181b levels could also be detected in isolated PBMCs and splenocytes, thus indicating the intracellular localization of the miRNA. In PBMCs, miR-181b returned to basal levels within 24 h. In liver and spleen, miR-181b returned to basal levels within 72 hours.

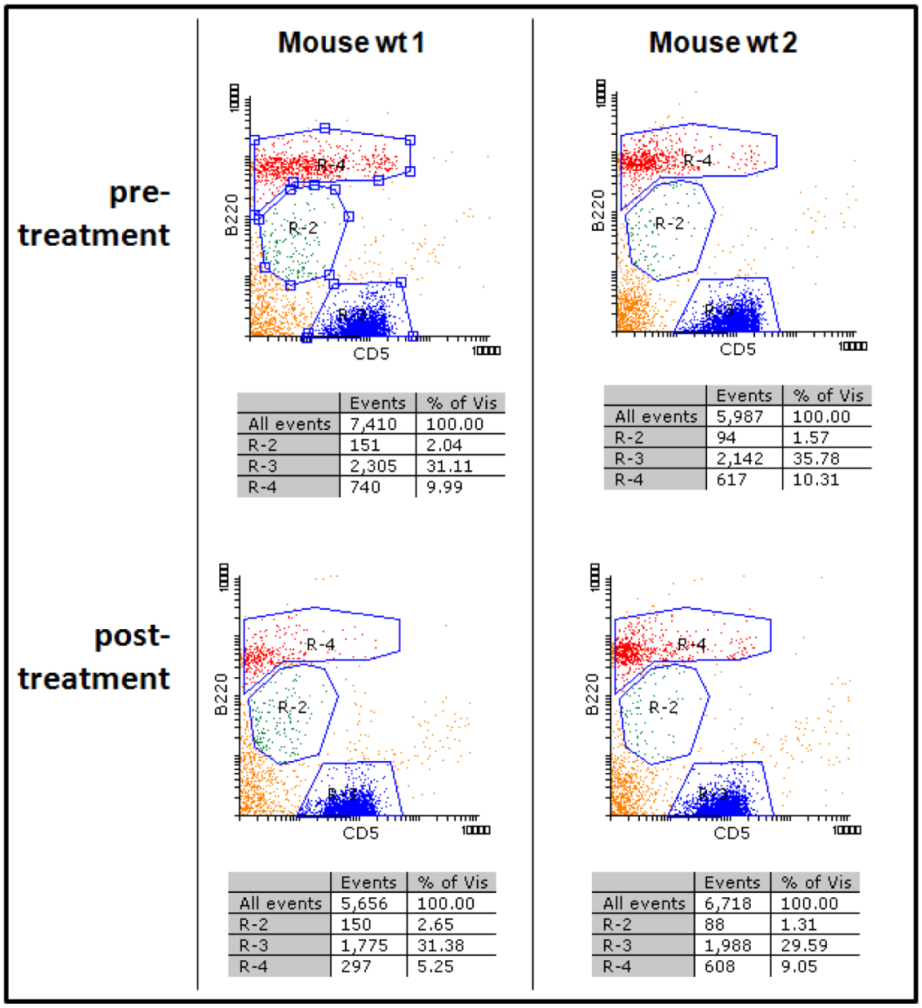

Mouse wt 1, pre-treatment

	Events	% of Vis
All events	7,410	100.00
R-2	151	2.04
R-3	2,305	31.11
R-4	740	9.99

Mouse wt 2, pre-treatment

	Events	% of Vis
All events	5,987	100.00
R-2	94	1.57
R-3	2,142	35.78
R-4	617	10.31

Mouse wt 1, post-treatment

	Events	% of Vis
All events	5,656	100.00
R-2	150	2.65
R-3	1,775	31.38
R-4	297	5.25

Mouse wt 2, post-treatment

	Events	% of Vis
All events	6,718	100.00
R-2	88	1.31
R-3	1,988	29.59
R-4	608	9.05

Figure S6. miR-181b treatment has a weak effect on viability of normal B and T cells. Following canonical 3 weeks of treatments, the pattern of B220/CD5 blood cell populations from two wild type mice (wt1 and wt2) were investigated. Compared to pre-treatment, there was a weak decrease of B220+/CD5- as well as B220-/CD5+ cells, suggesting that the treatment could minimally affect viability of these normal cell populations.
